# Time to remind us that absence of evidence is not evidence of absence during the coronavirus disease 2019 (COVID-19) pandemic

**DOI:** 10.1017/ice.2021.47

**Published:** 2021-02-04

**Authors:** Chenyu Sun, Ce Cheng, Mubashir Ayaz Ahmed, Qin Zhou

**Affiliations:** 1 AMITA Health Saint Joseph Hospital Chicago, Chicago, Illinois; 2 The University of Arizona College of Medicine at South Campus, Tucson, Arizona; 3 Radiation Oncology, Mayo Clinic, Rochester, Minnesota

*To the Editor*—During the current coronavirus disease 2019 (COVID-19) pandemic, guidelines issued by various agencies, including the US Centers for Disease Control and Prevention (CDC), have been conflicting on the issue of respiratory protection with a face mask or a respirator.^1^ The CDC has not officially announced the protective effects of masks for the wearers until recently,^2^ and even now, its web pages still show that surgical mask “is not considered respiratory protection.”^3^

Earlier this year, research on the protective effects of masks was limited, indicating lack of sufficient evidence to support the protective effects of masks to severe acute respiratory syndrome coronavirus 2 (SAS-CoV-2). However, the lack of evidence that masks have protective effects to respiratory viral infections is not equivalent to evidence that masks lack protective effects. It would be prudent to refrain from premature conclusions without further comprehensive studies. As Mark Twain said, “It ain’t what you don’t know that gets you into trouble. It’s what you know for sure that just ain’t so.” At this time, we remind ourselves that the absence of evidence is not evidence of absence.^4^ Interestingly, while evidence of mask use against other viruses has not been strong enough for the CDC to suggest the protective effects of mask wearing,^5^ remdesivir was approved for emergency or experimental use, with only limited evidence, as a therapeutic candidate due to its ability to inhibit SARS-CoV-2 in vitro and against other coronaviruses.^6^ When it comes to wearing masks, a simple nonpharmaceutical intervention method with minimal side effects, how did the lack of evidence lead to recommendations against wearing them among the general public at the beginning of the pandemic? And even later, the CDC only recommended wearing masks to prevent asymptotic carriers and presymptomatic patients from spreading the virus.^7^

Again, we remind ourselves, when issues of public health are concerned, we must question whether the absence of evidence is a valid justification for inaction.^8^ Statements about the absence of evidence are common, such as protective effects of masks for the general public at the beginning of current COVID-19 pandemic. However, can we be comfortable that the absence of solid and clear evidence is equivalent to the position that masks provide no protective effects or only negligible effects? For this global threat, it is better to be safe than sorry, and we should take every possible reasonable intervention.
